# The Fracture Resistance of Additively Manufactured Monolithic Zirconia vs. Bi-Layered Alumina Toughened Zirconia Crowns When Cemented to Zirconia Abutments. Evaluating the Potential of 3D Printing of Ceramic Crowns: An In Vitro Study

**DOI:** 10.3390/dj9100115

**Published:** 2021-10-08

**Authors:** Amirali Zandinejad, Marta Revilla-León, Mohammad Mujtaba Methani, Leila Nasiry Khanlar, Dean Morton

**Affiliations:** 1 Department of Comprehensive Dentistry, College of Dentistry, Texas A&M University, Dallas, TX 75246, USA; revillaleon@tamu.edu; 2 Department of Biomedical Sciences, College of Dentistry, Texas A&M University, Dallas, TX 75246, USA; metha010@umn.edu; 3 Department of Cariology and Operative Dentistry, Graduate School of Medical and Dental Sciences, Tokyo Medical and Dental University, 1-5-45, Bunkyo-ku Yushima, Tokyo 113-8510, Japan; lnasirykhanlar@gmail.com; 4 Department of Prosthodontics, Indiana University School of Dentistry, Indianapolis, IN 46202, USA; deamorto@iu.edu

**Keywords:** additive manufacturing, zirconia crown, alumina reinforced zirconia crown, fracture resistance

## Abstract

(1) Background: This study compared the fracture resistance of additively manufactured monolithic zirconia and bi-layered alumina toughened zirconia crowns on implants. (2) Methods: Maxillary model with a dental implant replacing right second bicuspid was obtained. Custom abutments and full-contour crowns for additively manufactured monolithic zirconia and bi-layered alumina reinforced zirconia crowns (*n* = 10) were fabricated. The crowns were cemented to implant-supported zirconia abutments and the assembly fixed onto resin blocks. Fracture resistance was measured using a universal testing machine at a crosshead speed of 2 mm/min. A Kruskal–Wallis test was used to analyze the data. (3) Results: Although additively manufactured monolithic zirconia crowns demonstrated a higher mean fracture resistance than bi-layered alumina toughened zirconia crowns, statistical analysis revealed no significant difference in fracture resistance between the two groups. All specimens fractured at the implant–abutment interface. (4) Conclusions: Additively manufactured bi-layered alumina toughened zirconia crowns demonstrated similar fracture resistance to additively manufactured monolithic zirconia crowns when cemented to implant-supported zirconia abutments.

## 1. Introduction

Due to their optical properties, ceramics have long been used to mimic the appearance of natural teeth in dental restorations [1]. Since the evolution of metal ceramic restorations more than 35 years ago, there have been several advances with regards to the mechanical properties and fabrication methods of all ceramic restorations in order to enhance esthetics by replacing the metal core [2,3]. Even though all ceramic restorations impart a phenomenal combination of biocompatibility and esthetics, different studies have demonstrated their higher incidence of fracture when compared to conventional metal–ceramic prostheses. Their higher tendency to develop fractures could be related to their brittleness [4,5].

Increasing demands for esthetic dentistry and tooth color restorations have led to utilizing ceramics in implant dentistry mostly for replacing missing teeth in the esthetic zone [6,7]. Ceramic abutments were developed to optimize the esthetic outcome in the esthetic zone with respect to final color of the restoration and soft tissue surrounding the crown [8,9,10]. However, all ceramic crowns, especially when supported by implants, will be more prone to fracture under occlusal forces [11,12].

Interestingly, contrasting the layered tooth structure, i.e., enamel and dentin, with other multilayer systems, such as porcelain fused to metal restorations or all ceramic restorations, reveals that a natural tooth has less chipping or cracking problem [13,14]. Unique structural combination of human enamel and dentin could be the reason behind the long-term survival of this system [15,16,17,18,19,20]. Recently, it has been demonstrated that enamel and dentin are not confined to a homogeneous structure. In fact, both exhibit a graded structural design. In a very recent study by He et al., enamel shows a decreasing elastic modulus and hardness from cusp tips to dentin–enamel junction (DEJ). The graded enamel is better adapted to stress distribution in the enamel and along the DEJ [19]. Zhang and co-workers fabricated graded structures by infiltrating glass into zirconia plates and demonstrated a significant increase in the fracture loads of the infiltrated material [20].

Milling or subtractive manufacturing is the state-of-the-art technology to manufacture all-ceramic restorations, such as zirconia [21]. However, the limitations of subtractive technology include wastage of material, introduction of microcracks, and limitation to fabricate complex geometries [22,23].

Additive manufacturing (AM) technologies also known as 3D printing are an alternative to milling for the fabrication of dental devices, mainly resin and metal prosthesis [24,25,26] with limited progress in fabrication of zirconia and ceramics [27,28,29]. Additive manufacturing has been defined by the American society of Testing and Materials as “the process of joining materials to make objects from 3-dimensional (3D) model data, usually layer upon layer, as opposed to subtractive manufacturing methodologies” [30]. AM has many advantages including being able to create dental restorations with complex macro geometries and controlled gradients [30,31].

Additive manufacturing enjoys several advantages over subtractive manufacturing, including fabrication of complex geometries and the ability to form structures in multiple materials. All the rapid prototyping techniques are based on similar premises. It has been demonstrated that by using different fabrication parameters, rapid prototyping can produce both fully sintered (solid) and partially sintered (more porous) structures. Accordingly, it is possible to utilize this process to create dental restorations with complex macro geometries and controlled gradient porosities, which cannot be fabricated using conventional machining technique. Therefore, AM potentially allows for the fabrication of functionally graded dental restorations emulating the mechanical properties of human enamel and dentin [32,33,34,35,36,37].

The objective of the present in vitro study was to fabricate bi-layered all-ceramic dental crowns with zirconia and alumina toughened zirconia using additive manufacturing technologies and to compare the fracture resistance of bi-layered alumina toughened zirconia (AMAlZr) crowns with additively manufactured monolithic zirconia crowns (AMZr) when cemented to milled zirconia implant abutments. The null hypothesis would be that there are no significant differences in the fracture resistance of AM zirconia and AM bi-layered alumina toughened zirconia.

## 2. Materials and Methods

**Figure 1:**
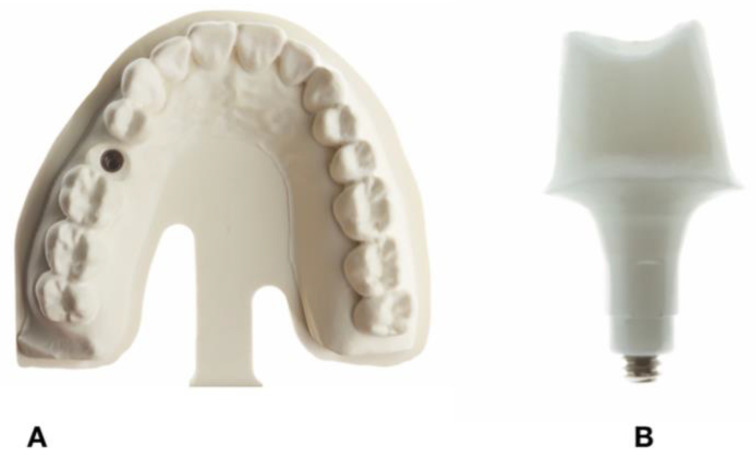
(**A**), Maxillary model with implant placed in right maxillary second bicuspid. (**B**), Milled Zirconia abutment.

A maxillary model with an implant replacing right second bicuspid representing a clinical scenario was selected (Figure 1A,B). The model was digitized using a dental laboratory scanner (DWOS 7 Series scanner; Straumann, Basel, Switzerland). The custom abutment with a chamfer finish line, buccal and lingual wall height of 6 mm, and a proximal wall height of 4 mm and a total convergence angle of 10 to 12 degrees (Figure 1A,B) was designed using CAD software (CARES Software; Straumann, Basel, Switzerland) and the Standard Tessellation Language (STL)_1_ file was used to manufacture zirconia implant abutments (CARES zirconium-dioxide abutment; Straumann, Arlington, TX, USA). A total of 20 zirconia abutments were milled.

**Figure 2:**
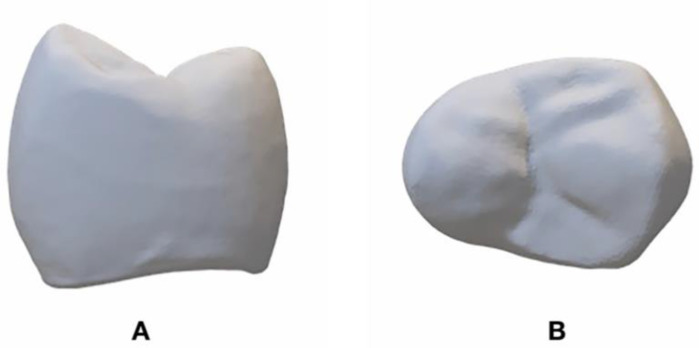
STL_2_ file. (**A**), proximal view. (**B**), occlusal view.

**Figure 3:**
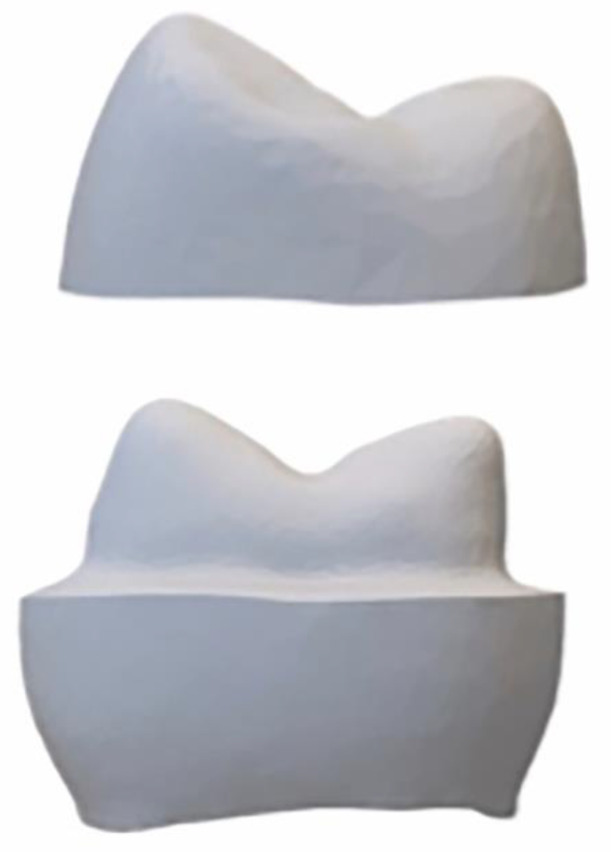
STL_2_ file split in top and bottom layers.

**Figure 4:**
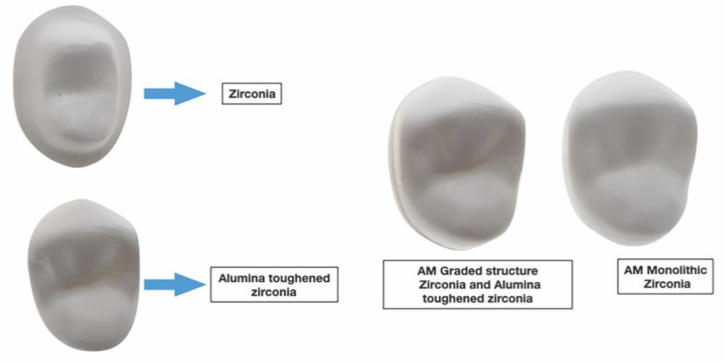
Additively manufactured Zirconia and bi-layered alumina reinforced zirconia.

A full contour crown was designed for the abutment using the same CAD software and the STL_2_ file (Figure 2A,B) was obtained. The thickness of the crown ranged from 1.0 mm (at the margin) to 2.0 mm (at the occlusal surface). The STL_2_ file was used to fabricate (CeraMaker 900; 3DCeram Co. Lemonge, France) 10 full-contour zirconia (3DMix ZrO_2_ paste; 3DCeram Co. Lemonge, France) crowns [38]. Thereafter, the STL_2_ file was split in thickness into 2 layers (Figure 3). The bottom layer facing the intaglio surface was AM in zirconia (Table 1) and the top layer harboring the occlusal surface was AM in Alumina toughened zirconia (ATZ) (Table 1). A count of 10 was manufactured for each component layer. Each bottom layer was cemented (Speedcem plus; Ivoclar Vivadent, Schaan, Liechtenstein) to its corresponding top layer to attain 10 samples of full contour premolar crowns printed to resemble the bi-layered configuration. However, we were unable to create a true simultaneous design due the limitations in AM ceramic technology. All the AM samples were produced by the manufacturer (3DCeram Co. Limonge, France) (Figure 4).

**Figure 5:**
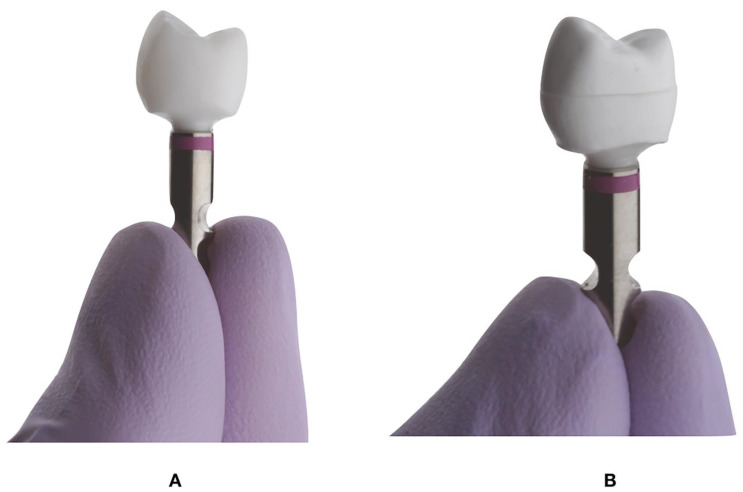
Crowns cemented to zirconia abutments before mechanical testing. (**A**), AM full contour Zirconia. (**B**), AM alumina toughened zirconia crown.

All the zirconia abutments were positioned on implant analog and torqued to 35 N/cm (Figure 5) (Straumann RC; Straumann, Basel, Switzerland) and divided into 2 groups: additively manufactured monolithic zirconia crowns (AMZr) and additively manufactured bi-layered alumina toughened zirconia crowns (AMAlZr) (Table 2).

The screw access was sealed with a teflon tape and all the abutments and the intaglio surfaces of the crowns in both groups (AMZr and AMAlZr) were cleaned (Ivoclean; Ivoclar Vivadent Schaan, Liechtenstein) following the manufacturer’s instruction. Subsequently, the crowns were cemented with a self-adhesive resin cement (Speedcem plus; Ivoclar Vivadent Schaan, Liechtenstein) on the abutments. The excess cement was cleaned using a 2 × 2 gauze, and all surfaces were cured with LED curing light (3M ESPE Elipar S10; 3M ESPE, 3M Co., St.Paul, MN, USA) for 20 s to ensure adequate polymerization [38].

A 12-mm deep hole was drilled into the center of cuboid polyurethane blocks (SKU: 1522-05, Saw Bones, Vashon WA, USA) for mounting the implant analogs, abutment, and crown assemblies (Figure 5A,B) using a resin cement (Methyl methacrylate Resin; Monomer-Polymer & Dajac Laboratories INC., Trevose, PA, USA). The cement was allowed to set for 24 h before subjecting the samples to mechanical loading [38].

A mandibular right second bicuspid Co-Cr crown was used as an antagonist to load the experimental crowns. It was cemented using resin cement (Methyl Methacrylate Resin; Monomer-Polymer & Dajac Laboratories INC. Trevose, PA, USA) on a Titanium rod. The assembly contributed to the loading arm and was mounted onto the loading frame of the universal testing machine (MTS Bionix 370; MTS Systems Corp.) [38].

Polyurethane blocks harboring the abutment and crown assemblies were affixed between two metal arms on the horizontal platform of the universal testing machine (MTS Bionix 370; MTS Systems Corp. Eden Prairie, MN, USA). The specimens and the loading metal crown were positioned into maximum intercuspation. All specimens were subjected to static vertical loading using the universal testing machine (MTS Bionix 370; MTS Systems Corp. Eden Prairie, MN, USA) at a crosshead speed of 2 mm/min and 25 kN load cell [38,39]. Force–displacement curves were recorded for all the specimens. Following the test, all the specimens were analyzed to determine the mode of failure [38].

A statistical software (SPPS v22; IBM Corp. Armonk, NY, USA) was used to calculate the means and standard deviations of the fracture resistance in both groups. The Mann Whitney U test was used to determine the existence of a significant difference, if any, in fracture resistance between the groups as the data were not normally distributed.

## 3. Results

**Figure 6:**
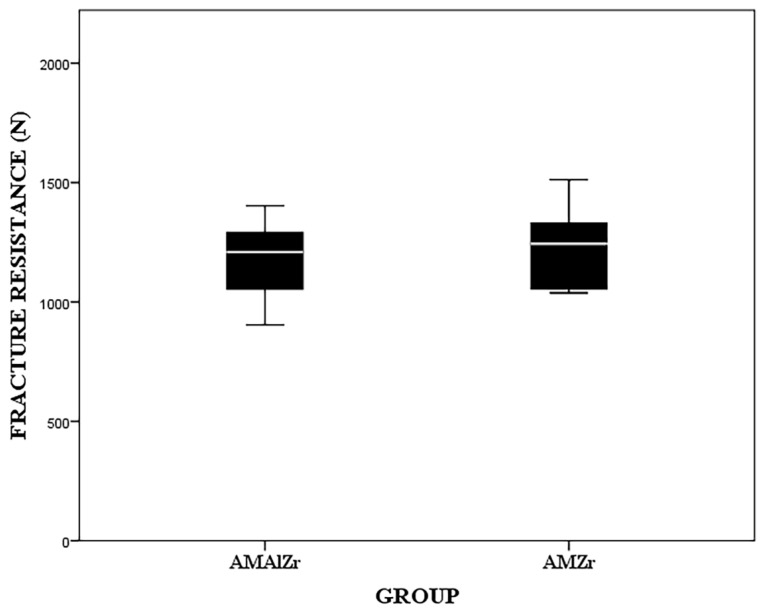
Box plot representing fracture resistance of AMZr and AMAlZr.

Although the AMZr crowns demonstrated a higher median fracture resistance (1243.5 ± 265.5 N) than AMAlZr (1209 ± 204.5 N) crowns (Figure 6), the Mann Whitney U test indicated that there was no significant difference in fracture resistance (*p* = 0.6) between the two groups.

**Figure 7:**
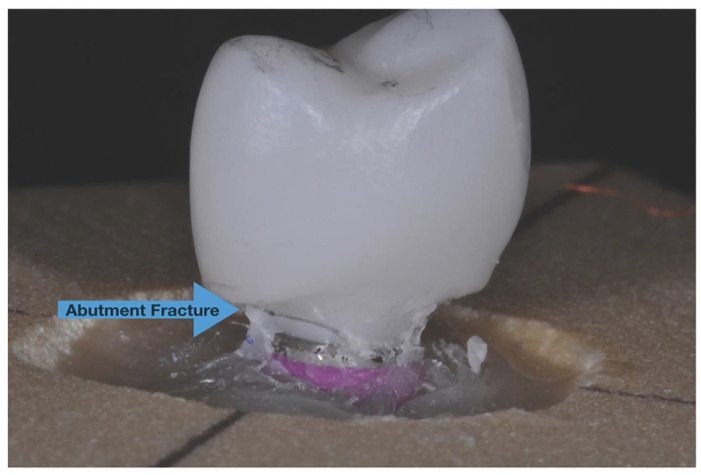
The fracture of zirconia abutment near the interface of zirconia abutment and implant analog which was recorded for all specimens in both groups.

Samples in both groups fractured at the abutment level near the interface of zirconia abutment and implant analog with no significant differences between two groups (Figure 7). The crowns were intact in both groups after the fracture resistance test.

## 4. Discussion

The bi-layered ceramic restorations were expected to demonstrate higher values for fracture resistance, owing to their tendency to mimic the structure of human enamel and dentin. The goal of this study was to evaluate the potential of additive manufacturing in fabricating a bi-layered design as a first step to create a multilayered graded structural design which represents human enamel and dentin [33,34,35,36]. However, AM technology was not able to fabricate bi-layered or multilayered graded structural ceramic restorations as expected in this study.

The concept of a bioinspired graded structure relies upon designing a restoration, such that it mimics the architecture of enamel and dentin in natural tooth. In that context, it means that the reduction in hardness and modulus of elasticity in a dental crown should reflect a continuous gradient from occlusal to the intaglio surface [19,37]. Although the concept has been described [36], AM technologies have not matured sufficiently to be able to imitate such a bio-inspired structure. Moreover, the limitation of ceramics available for 3D printing constrained us from selecting the appropriate materials required to duplicate the mechanical properties of enamel and dentin in bi-layered or graded design [19]. However, these problems are expected to resolve in the near future, following advances in the AM technology.

Zirconia abutments were used in this in vitro study instead of metal abutments, because of their superior esthetic for patients with a high lip line and thin gingival phenotype [8,9,10]. Although titanium abutments withstand significantly higher loads than zirconia abutments before fracturing [9,11], zirconia abutments are strong enough to withstand occlusal forces in the anterior region [40,41,42]. In a study by Martinez et al., the mean fracture resistance values of milled zirconia crowns cemented to zirconia abutments were 340.3 N [43]. In this study, the mean fracture resistance value for AMZr crowns was 1330 N. Although this study did not compare the fracture resistance of milled zirconia to that of AM zirconia, the authors of a very similar study found no significant differences between milled zirconia and AM zirconia [38].

Although AM offers many advantages, the AM of dental ceramics is not a valid and accepted fabrication technique yet. There have been very limited studies on 3D printing of dental ceramics with no published studies that have investigated the fracture resistance of 3D printed bi-layered alumina reinforced zirconia ceramic crowns supported by implants [28,44] to compare and validate the findings of this study.

Zirconia abutments were the common mode of failure for all the specimens after mechanical loading and the result is similar to a previous study by Martinez et al. [42] Using titanium abutments or a combination of zirconia with titanium base could have potentially changed the mode of failure by changing the weakest point, which was the zirconia abutment in this study.

Fracture of the veneering material, including porcelain chipping is the most common complication associated with implant-supported prostheses. This percentage was higher with all-ceramic crowns [12]. Collectively, the advantages of all ceramic restorations deem it essential to mitigate the complications associated with their clinical applications, particularly in implant dentistry. Additive manufacturing provides many advantages over milling which may enable us to overcome the existing limitations during manufacturing of ceramic restorations [35]. Based on the results of this study, AM of bi-layered alumina toughened zirconia crowns demonstrated a comparable fracture resistance to AM monolithic zirconia crowns when cemented to zirconia abutments, which in turn is not significantly different from milled zirconia crowns [38]. However, this is a pilot study and further investigation is necessary to validate the additive manufacturing of zirconia and alumina toughened zirconia as a viable technology for the fabrication of restorations in clinical dentistry.

## 5. Conclusions

Based on the experimental design and the limitations of the present study, no significant differences were encountered in fracture resistance between additively manufactured monolithic zirconia and bi-layered alumina toughened zirconia crowns. Based on the results obtained, AM appears to be a promising technology for fabricating zirconia and alumina toughened zirconia restorations with great potential for improvement in the near future. The expansion of the AM technology can incorporate the fabrication of ceramic based bio-inspired graded structural crowns as a treatment modality and allow for the exploration of their physical and mechanical properties.

## Figures and Tables

**Table 1:** Physical and mechanical properties of additive manufactured zirconia and alumina toughened zirconia material. Information provided by the manufacturer.

Physical/Mechanical Properties	3DMix ZrO_2_ 3D CERAM	3DMix ATZ 3D CERAM
Grade	700	NP *
Particle size (μm)	0.1–0.8	>5.2
Density (g/cm^3^)	5.97	>5.2
Vickers Hardness (GPa)	12.6	NP*
Young’s modulus (GPa)	209.4	220
Weibull modulus	NP *	5.8
Shear modulus (GPa)	79.8	NP *
Flexural strength (MPa)	1088	1094
Compressive strength (MPa)	2070	NP *
Coefficient thermal expansion (K^−1^)	12.4	7.50 to 8.33

* NP: Not provided.

**Table 2:** Characteristics of milled and stereolithography (SLA) additive manufactured (AM) zirconia specimens.

Group	Material	Fabrication Technique	Composition
AMZr	3DMix ZrO_2_ (3D Ceram)	Laser Stereolithography (SLA)	Zirconia stabilized with 3% yttria
AMAlZr	3DMix ATZ (3DCeram)	Laser Stereolithography (SLA)	The ceramic ATZ combines both Alumina (20%) and Zirconia (80%) ceramics in one

## Data Availability

Not applicable.
